# Radiation Reduction and Protection for Radiosensitive Organs (Lens, Thyroid, and Genital Organs) of Patients Receiving Percutaneous Coronary Intervention—Real-World Measurement of Radiation Dose in a Single Center

**DOI:** 10.3390/jcdd8080099

**Published:** 2021-08-20

**Authors:** Wen-Hwa Wang, Kai-Che Wei, Wei-Chun Huang, Yuan-Yin Yen, Guang-Yuan Mar

**Affiliations:** 1 Cardiovascular Center, Kaohsiung Veterans General Hospital, Kaohsiung 813, Taiwan; passtheboard1@gmail.com (W.-H.W.); wchuanglulu@gmail.com (W.-C.H.); yyyen@vghks.gov.tw (Y.-Y.Y.); 2 College of Management, I-Shou University, Kaohsiung 840, Taiwan; 3 Health Management Center, Kaohsiung Veterans General Hospital, Kaohsiung 813, Taiwan; 4 Division of Dermatology, Kaohsiung Veterans General Hospital, Kaohsiung 813, Taiwan; kaiche92@gmail.com; 5 School of Medicine, National Yang Ming Chiao Tung University, Taipei 112, Taiwan; 6 Department of Critical Care Medicine, Kaohsiung Veterans General Hospital, Kaohsiung 813, Taiwan; 7 Department of Physical Therapy, Fooyin University, Kaohsiung 831, Taiwan; 8 College of Health and Nursing, Meiho University, Pingtung 912, Taiwan; 9 Superintendent, Kaohsiung Municipal United Hospital, Kaohsiung 804, Taiwan

**Keywords:** Percutaneous Coronary Intervention (PCI), radiation injury, radiation protection, frame rate of cineangiographic images

## Abstract

Backgrounds: Reducing radiation exposure is the basic principle for performing percutaneous coronary intervention (PCI). Many studies have confirmed the effect of radiation protection for medical staff, but studies about the effectiveness of protection for patients and real measurement of radiation dose in patients’ specific organs are lacking. Aim: To measure the radiation doses absorbed by patients’ radiosensitive organs during PCI and the effectiveness of radiation protection. Methods: A total of 120 patients were included and allocated into three groups as the ratio of 1:1:2. A total of 30 patients received PCI at 15 frames rate per second (fps), 30 patients at 7.5 fps, and 60 patients wore radiation protective hat and glasses during PCI at 7.5 fps. The radiation doses were measured at right eyebrow (lens), neck (thyroid), back (skin), and inguinal area (genital organs) by using thermoluminescent dosimeters (TLDs). Results: Dose-area product (DAP) reduced by 58.8% (from 534,454 ± 344,660 to 220,352 ± 164,101 mGy·cm^2^, *p* < 0.001) after reducing the frame rate, without affecting successful rate of PCI. Radiation doses measured on skin, lens, genital organs, and thyroid decreased by 73.3%, 40.0%, 40.0%, and 35.3%, respectively (from 192.58 ± 349.45 to 51.10 ± 59.21; 5.29 ± 4.27 to 3.16 ± 2.73; 0.25 ± 0.15 to 0.15 ± 0.15; and 17.42 ± 12.11 to 11.27 ± 8.52 μSv, *p* < 0.05). By providing radiation protective equipment, radiation doses at lens and thyroid decreased further by 71.8% and 65.9% (from 3.16 ± 2.73 to 0.89 ± 0.79; 11.27 ± 8.52 to 3.84 ± 3.49 μSv, *p* < 0.05). Conclusions: By lowering the frame rate and providing protective equipment, radiation exposure in radiosensitive organs can be effectively reduced in patients.

## 1. Introduction

Cardiovascular disease is one of the leading causes of death globally and is the second most common cause of death in Taiwan [1]. Percutaneous coronary intervention (PCI), with approximately one million cases per year in the U.S.A., utilizes radiation to visualize coronary arteries and has been the primary treatment for coronary artery disease. However, due to the improvement of PCI devices and techniques, more interventional cardiologists tend to challenge increasingly complex cases, such as chronic total occlusion lesions. Additionally, due to multiple lesions of coronary arteries and in-stent restenosis, numerous patients have undergone repetitive PCIs. Therefore, more patients are being exposed to higher doses during PCI procedures than they were decades ago. 

While performing PCI, the skin of the patient’s upper back usually receives the largest radiation dose. Not only the main beam of radiation, scattered radiation causes hazards to other radiosensitive organs, such as thyroid, eye lens, and gonads. According to the International Commission on Radiation Protection (ICRP), no “minimum safe radiation dose” for development of cancer has been established [2,3]. Despite the dose of radiation exposure during PCI rarely exceed the threshold of causing immediate clinical damage, our study recently demonstrates the radiation of PCI is associated with the increased risks for lens surgery for cataract [4]. It is very likely that no matter how low the radiation dose is, the patient’s DNA may be damaged. The consequence is an increase in the incidence of radiation associated damage, such as lens opacity and malignancies. Therefore, reducing radiation exposure as much as possible is the mandatory principle while performing PCI. According to guidelines of performing PCI [5], interventional cardiologists and catheterization lab staff are advised to reduce the radiation exposure of radiosensitive organs as well as record the radiation dose in every procedure. Furthermore, avoiding unnecessary radiation exposure and putting protection equipment outside of the operative view can further reduce radiation dose. 

Since patient safety is the most important concern for physicians, it is necessary to prevent patients from being injured by radiation. Radiation reduction can be done in many ways, such as administering radiation education to interventional cardiologists as well as catheterization lab staff [6]. One ideal method is renewal of the X-ray equipment in catheterization labs by purchasing the latest machines, up-to-date software, and real-time dose monitoring [7,8]. However, financial constraints limit accessibility for many catheterization labs. In reality, it is more feasible to use a strategy with lowering frame rate and providing patients with radiation protective equipment during PCI. However, there are scarce evidences to provide real-world data about effect of routine radiation reduction and protection in PCI patients. Therefore, we conducted this perspective real-world study, to directly measure exposed dose of radiation in patients’ radiosensitive organs, and assess the influence of a frame rate lowering strategy and wearing protective equipment.

## 2. Materials and Methods

### 2.1. Study Design

This study followed the guidelines of the Declaration of Helsinki. The Ethics Committee and the Institutional Review Board of Kaohsiung Veterans General Hospital approved this study (IRB number: VGHKS16-CT8-04) and permitted the informed consent for study participation to be waived.

This study was performed by a multidisciplinary team in a single medical center from June 2017 to October 2019. This study was conducted in a single catheterization lab using the fluoroscopic radiator (Clarity IQ, Philip Allura Xper FD 10/10, the Netherlands). Patients with coronary artery disease were randomly chosen for the placement of radiation detectors upon receiving the scheduled percutaneous coronary intervention. 

The radial approach has been preferred while performing PCI, and was more frequently used in this study [9]. The right radial artery (RRA) was the default access, and the femoral artery was used in some cases of acute coronary syndrome (ACS), chronic total occlusion (CTO), or if RRA was clinically not feasible. Although no fixed protocolized views were defined, a minimum of four to six views were obtained to image the left coronary system and two to three views were obtained for the right coronary artery in diagnostic coronary angiogram and adequate projections of target lesion PCI was dependent on the operator’s choice.

**Figure 1:**
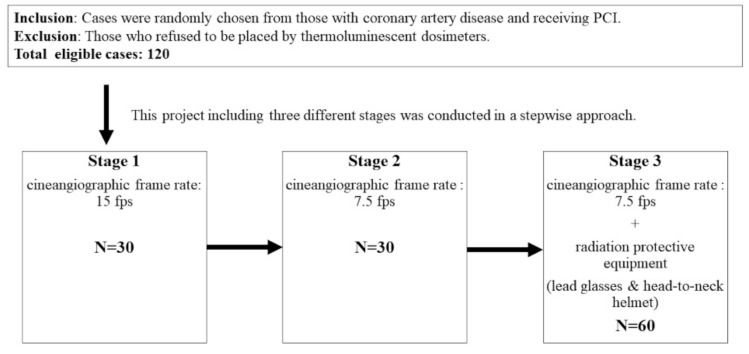
The study flow-chart. This study included patients who were admitted for PCI. Those who refused to be placed on thermoluminescent dosimeters were excluded. After coronary angiography, those for whom PCI was not recommended would be excluded from this study.

The study was composed of three different stages and conducted in a step-by-step manner. The graphic flow-chart was illustrated in Figure 1. In the first stage, the frame rate of cineangiographic images was set at 15 frames/second (fps). In the second stage, the low frame rate (7.5 fps) strategy was used. In the third stage, in addition to the low frame rate strategy (7.5 fps), the patients were provided with radiation protective equipment without interfering with the PCI views (Supplementary Figure S1). This radiation protective equipment was composed of lead eyeglasses (0.5 mm Pb equivalent) and radiation protective head-to-neck helmets (0.5 mm Pb equivalent), which covered the entire head and neck (Supplementary Figure S1). The person in the figure provided a written informed consent. In stage 2 and 3, the interventional cardiologists could require to switch to a higher frame rate while dealing with complex lesion, and were encouraged to switch back to a lower frame rate after treating the complex lesion.

There were no previous and relevant studies to provide to calculate the minimal clinically relevant difference between stages I and II or stages II and III, instead, we used effect size Cohen’s d to calculate the sample size. We assumed the low frame rate strategy and the radiation protective equipment performed in stages II and III could substantially decrease the dose area production and the measured radiation dose over the eyebrow, respectively. We estimated that with 52 patients (26 and 26 patients in stages 1 and 2) and 58 patients (19 and 39 patients in stages 2 and 3), the trial would have at least 80% power to statistically detect a large effect size of Cohen’s d 0.8 at a two-sided significance level of 0.05 with G*power software. To meet the minimal requirement of each stage concurrently, we estimated 120 patients needed to be included (30, 30, and 60 patients in stages 1, 2, and 3).

### 2.2. Measurement

The overall fluoroscopy time, radiation output, and dose-area product (DAP; mGy·cm^2^) were recorded. Organ-specific radiation dose was measured with thermoluminescent dosimeter (TLD) (Becquerel and Sievert, Taipei, Taiwan. License number of Taiwan Atomic Energy Council: L0622-180123). TLDs were placed on the skin of the lower back (at the level of T-L spine), mid neck (above thyroid), left inner thigh (close to genital organs), and on the right eyebrow (close to eye lens). The exposed radiation doses (μSv) were recorded as Hp (10) and Hp (0.07), which were used to estimate the dose equivalent in soft tissue at a depth of 10 mm and 0.07 mm.

### 2.3. Statistical Analysis

The collected data were analyzed using SPSS ver. 20 (IBM Corp., Armonk, NY, USA). Differences between the parameters for the three patient groups were tested by a one-way ANOVA test, and a Student’s t-test was used to compare the two groups. A *p*-value < 0.05 was considered to be statistically significant.

## 3. Results

There were 30, 30, and 60 patients in stages I, II, and III, respectively. The basic characteristics of the three groups as well as the distribution for the targeted coronary arteries were not significantly different. There were no differences in the prevalence of bleeding or major adverse cardiovascular events (MACE) following PCI (Table 1). Reducing the frame rate from 15 fps to 7.5 fps did not prolong the overall fluoroscopy time (stage I: 28.76 ± 17.92, stage II: 31.25 ± 14.72, and stage III: 35.67 ± 30.85 minutes; *p* = 0.429) (Table 1) or lower the success rate of these procedures.

**Figure 2:**
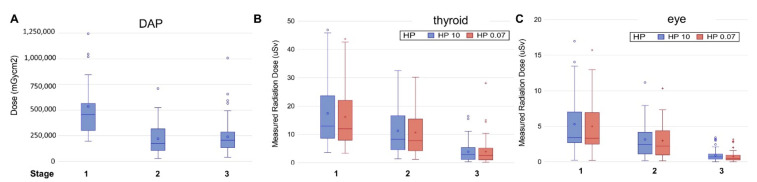
The distribution of (**A**) radiation output during PCI, (**B**) received radiation dose on neck (thyroid), and (**C**) right eye (lens) in stages 1, 2, and 3. (**Stage 1**: frame rate of 15 frames/second, without protection; **Stage 2**: 7.5 frames/second, without protection; and **Stage 3**: 7.5 frames/second, with protection; **DAP**: Dose Area Production).

We compared the changes of DAP (mGy·cm^2^) and radiation dose (μSv) in different radiosensitive organs. By reducing the frame rate from 15 fps (stages I) to 7.5 fps (stages II and III), the overall magnitude of reduction in DAP was 58.8% and 55.3% (from stage I: 534,454 ± 344,660 mGy·cm^2^, to stage II: 220,352 ± 164,101 mGy·cm^2^, and stage III: 238,969 ± 175,064 mGy·cm^2^, *p* < 0.001) (Figure 2A). 

Comparing stages I and II, the measured received radiation dose decreased concordantly, over eyebrow (40.0%, from 5.29 ± 4.27 to 3.16 ± 2.73 μSv), mid back (73.3%, from 192.58 ± 349.45 to 51.10 ± 59.21 μSv), neck (by 35.3%, from 17.42 ± 12.11 to 11.27 ± 8.52 μSv), and inner thigh (by 40.0%, from 0.25 ± 0.15 to 0.15 ± 0.15 μSv; all *p* < 0.05) (Table 2).

In the third stage, patients were provided with lead glasses and a radiation protective head-to-neck soft helmet for protection of eyes and thyroid. None of the patients reported any feelings of discomfort while wearing the lead head-to-neck helmet since the material was soft and breathable on the top of the head. No patient had asked to remove the lead head-to-neck helmet during the procedure. The radiation dose was further decreased by 71.8% on the eyebrow (Hp (10), changed from 3.16 ± 2.73 to 0.89 ± 0.79 μSv, *p* < 0.001), and by 65.9% on the neck (Hp (10), changed from 11.27 ± 8.52 to 3.84 ± 3.49 μSv, *p* < 0.001) (Table 2) (Figure 2B,C). As expected, the radiation doses measured on the unprotected area (back skin and inner thigh) did not change significantly.

## 4. Discussion

Our study demonstrates a real-world evidence supporting that both a frame rate lowering strategy and wearing protective equipment significantly reduce radiation exposure in radiosensitive organs (eye, thyroid, and reproductive organs) in patients receiving PCI.

Patient safety cannot be overemphasized. All cardiologists and catheterization lab staff should strive to lower the harm to their patients as much as possible. This study demonstrated that reducing the frame rate can effectively decrease the radiation dose received by patients, which is compatible with previous literature. Plourde et al. revealed that frame rate reduction, selective fluoroscopy storage, and renewal of X-ray equipment assisted in lowering radiation exposure [10,11]. Visualization magnification is also an important factor affecting the overall radiation output. Hence, interventionists should try to use the lowest magnification and avoid using unnecessary high magnification. However, in practice, it is impractical to reduce radiation emission too much during a PCI procedure since the interventional cardiologists may not be able to see the images clearly. It is essential to switch to higher frame rate while treating high risk or complex lesions for the best results. Therefore, there should be a balance between image quality and radiation emission in order to perform procedures successfully. In this study, there were no differences in the PCI success rate, procedure time, or MACEs among the three study groups, which proves that a frame rate reducing strategy does not affect the PCI success rate. 

The use of proper radiation protection equipment without interfering with PCI performance can be a promising method for decreasing harm caused by ionizing radiation. To our knowledge, there are limited studies about directly measuring exposed before and after radiation protection for patients during PCI. In our study, by wearing proper radiation protective equipment to patients, patients received a lower radiation dose without jeopardizing the success of PCI. The protective lead head-to neck helmet, with the addition of lead glasses, is able to protect the whole brain, thyroid, and eye lens from the main radiation beam located under the table and the scattered radiation from all directions. Furthermore, it is cost-effective, since the protective head-to-neck helmet and lead eyeglasses cost roughly 300 US dollars and they are reusable after being appropriately sterilized.

Although the measured exposed radiation in the reproductive organs was very low in our study, it is ideal to further minimize the radiation. But, protection for the reproductive system is difficult and is not conclusive in previous literature. One study even showed that there was a higher total radiation exposure for patients utilizing an apron covering the genital area [12]. We assume if the apron blocks the radiation visualization field, the radiator may automatically increase the frequency of the radiation rate to ensure the image quality, which results in an increased total radiation.

## 5. Study Limitation

This is a single medical center with a relatively small case number and was conducted with a Philip X system, not a multicenter design with many different brands of radiators. Next, different radiation projection angles were used for different locations of coronary atherosclerosis lesions, which can affect the radiation output rate and the fraction of received radiation by different organs. Different magnifications also affect the frequency of radiation output. Ideally, a fixed radiation output with the same projection angle and the same magnification should be adopted in a study; however, this is not feasible in daily practice since this study is a real-world design. This study didn’t include many ACS patients due to lack of time on explaining protocol, getting consent, and accurately placing TLD. However, due to the positive results of the study, we would improve the protocol in order to apply it to ACS patients in the future. 

## 6. Conclusions

Using a frame rate lowering strategy as well as wearing protective equipment are effective in reducing radiation exposure for patients receiving PCI.

## Figures and Tables

**Table 1:** Basic characteristics of patients at different stages.

	Stage I	Stage II	Stage III	*p*-Value
	15NP (N = 30)	7.5NP (N = 30)	7.5P (N = 60)	
Gender (M/F)	22/8	27/3	53/7	0.116
Age (y/o)	62.87 ± 12.82	59.20 ± 12.02	65.25 ± 10.85	0.071
Height (cm)	164.92 ± 9.66	167.23 ± 9.71	165.91 ± 7.48	0.583
Weight (kg)	73.03 ± 16.80	72.95 ± 14.54	73.34 ± 12.76	0.991
BMI	26.69 ± 4.09	26.00 ± 4.04	26.29 ± 3.47	0.775
Diabetes Mellitus	18 (60.0%)	9 (30.0%)	22 (36.7%)	0.040 *
Hypertension	25 (83.3%)	23 (76.7%)	44 (74.6%)	0.644
Dyslipidemia	18 (60.0%)	19 (63.3%)	45 (75.0%)	0.281
PCI indication				
ACS	2 (6.7%)	5 (16.7%)	7 (11.7%)	0.483
STEMI	0 (0.0%)	1 (3.3%)	0 (0.0%)	0.220
NSTEMI	1 (3.3%)	1 (3.3%)	4 (6.7%)	0.704
Unstable angina	1 (3.3%)	3 (10.0%)	3 (5.0%)	0.505
Stable Angina	28 (93.3%)	25 (83.3%)	53 (88.3%)	0.483
Access				
Radial	26 (86.7%)	23 (76.7%)	45 (75.0%)	0.434
Femoral	4 (13.3%)	5 (16.7%)	9 (15.0%)	0.937
Both	0 (0.0%)	2 (6.7%)	6 (10.0%)	0.200
PCI targeted vessel §				
LAD/LM	19 (63.3%)	20 (66.7%)	31 (51.7%)	0.323
LCx	12 (40.0%)	6 (20.0%)	16 (26.7%)	0.210
RCA	11 (36.7%)	10 (33.3%)	33 (55.0%)	0.086
PCI targeted vessel no.				
1	19 (63.3%)	25 (83.3%)	42 (70.0%)	0.210
2	10 (33.3%)	3 (10.0%)	16 (26.7%)	0.088
3	1 (3.3%)	2 (6.7%)	2 (3.3%)	0.731
Case number of CTO	3 (10.0%)	2 (6.7%)	9 (15.0%)	0.483
I.I. FoV				
25cm	27 (90.0%)	29 (96.7%)	52 (86.7%)	0.329
20cm	3 (10.0%)	1 (3.3%)	8 (13.3%)	0.329
Imaging Guide				
No	16 (53.3%)	14 (46.7%)	36 (60.0%)	0.477
IVUS	5 (16.7%)	2 (6.7%)	8 (13.3%)	0.485
OCT	6 (20.0%)	13 (43.3%)	16 (26.7%)	0.116
FFR	3 (10.0%)	1 (3.3%)	0 (0.0%)	0.045
MACE within 30 days				
Death	1 (3.3%)	1 (3.3%)	1 (1.7%)	0.843
Nonfatal MI	1 (3.3%)	1 (3.3%)	0 (0.0%)	0.362
Bleeding	0 (0.0%)	0 (0.0%)	1 (1.7%)	0.604
CABG within 30 days	0 (0.0%)	1 (3.3%)	1 (1.7%)	0.601
Fluoroscopy time (min)	28.76 17.92	31.25 ±14.72	35.67 ± 30.85	0.429
DAP Dose (mGy·cm^2^)	534,454 ± 344,660	220,352 ± 164,101	238,969 ± 175,064	<0.001 ** (15NP > 7.5NP) <0.001 ** (15NP > 7.5P)

**15NP**: 15 frames/second, without protection; **7.5NP**: 7.5 frames/second without protection; and **7.5P**: 7.5 frames/second, with protection; **ACS**: acute coronary syndrome; **CTO**: chronic total occlusion; **DAP**: Dose area production; **FFR**: fractional flow reserve; **I.I. FoV**: image intensifier Field of view; **IVUS**: intravascular ultrasound; **LAD**: left anterior descending artery; **LCx**: left circumflex artery; **LM**: left main coronary artery; **MACE**: Major adverse cardiovascular events (cardiovascular death, myocardial infarction, or ischemic stroke); **OCT**: optical coherence tomography; and **RCA**: right coronary artery (* *p* < 0.05, ** *p* < 0.01). § Some patients had received PCI for more than multiple lesions, thus the cumulative percentage exceeds 100%.

**Table 2:** Measured radiation dose (μSv) over the eye, mid neck, back skin, and genital area at three different stages.

	Stage I (15NP)	Stage II (7.5NP)	Stage III (7.5P)	15NP > 7.5NP *p*-Value	7.5NP > 7.5P *p*-Value
**Eye**					
HP (10)	5.29 ± 4.27	3.16 ± 2.73	0.89 ± 0.79	0.026 *	<0.001 **
HP (0.07)	5.00 ± 3.96	2.97 ± 2.55	0.73 ± 0.75	0.022 *	<0.001 **
**Mid neck**					
HP (10)	17.42 ± 12.11	11.27 ± 8.52	3.84 ± 3.49	0.027 *	<0.001 **
HP (0.07)	16.18 ± 11.27	10.68 ± 8.28	3.90 ±4.55	0.036 *	<0.001 **
**Back skin**					
HP (10)	192.58 ± 349.45	51.10 ± 59.21	59.01 ± 119.38	0.037 *	0.746
HP (0.07)	179.30 ± 325.45	53.61 ± 71.85	54.88 ± 111.22	0.047 *	0.955
**Genital area**					
HP (10)	0.25 ± 0.15	0.15 ± 0.15	0.10 ± 0.15	0.016 *	0.085
HP (0.07)	0.19 ± 0.13	0.13 ± 0.14	0.06 ± 0.13	0.076	0.031 *

**15NP**: 15 frames/second, without protection; **7.5NP**: 7.5 frames/second without protection; and **7.5P**: 7.5 frames/second, with protection; * *p* < 0.05, ** *p* < 0.01.

## Supplementary Materials

The following are available online at https://www.mdpi.com/article/10.3390/jcdd8080099/s1, Supplementary Figure S1: The head-and-neck helmet and lead eyeglasses.
