# Supplementary material for: Radiation Reduction and Protection for Radiosensitive Organs (Lens, Thyroid, and Genital Organs) of Patients Receiving Percutaneous Coronary Intervention—Real-World Measurement of Radiation Dose in a Single Center
Source: J Cardiovasc Dev Dis. 2021 Aug 20;8(8):99. doi: 10.3390/jcdd8080099 (PMC8396847; doi:10.3390/jcdd8080099)

Supplementary figure

This radiation protective equipment is a soft cap composed of lead eyeglasses (0.5 mm Pb equivalent) and radiation protective head-to-neck helmets (0.5 mm Pb equivalent), which covered the entire head and neck (supplementary figure). The person in the figure is one of the coauthors of this article and has provided a written informed consent.

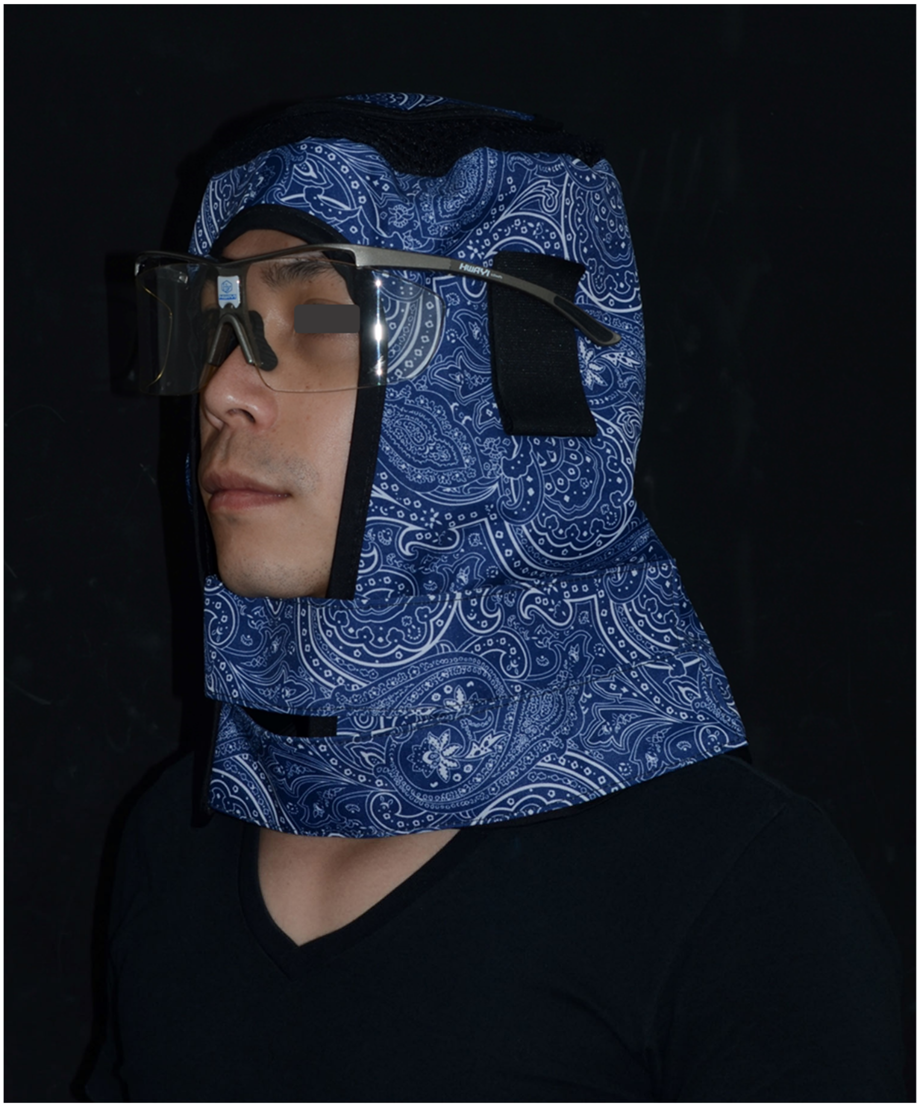

Supplement: Supplementary file 1 [file jcdd-08-00099-s001.zip › jcdd-1314487-supplementary.pdf]
